# Preparation of *Escherichia coli* ghost of anchoring bovine *Pasteurella multocida* OmpH and its immunoprotective effect

**DOI:** 10.1186/s12917-023-03743-9

**Published:** 2023-10-06

**Authors:** Nannan Chen, Dongjun Jiang, Yu Liu, Zecai Zhang, Yulong Zhou, Zhanbo Zhu

**Affiliations:** 1 College of Animal Science and Veterinary Medicine, Heilongjiang Bayi Agricultural University, Daqing, 163319 China; 2 Key Laboratory of Bovine Disease Control in Northeast China, Ministry of Agriculture and Rural Affairs, Daqing, 163319 China; 3 Shandong Collaborative Innovation Center for Diagnosis, Treatment and Behavioral Interventions of Mental Disorders, Institute of Mental Health, Jining Medical University, Jining, 272067 China; 4 Shandong Key Laboratory of Behavioral Medicine, School of Mental Health, Jining Medical University, Jining, 272067 China; 5 Engineering Research Center for Prevention and Control of Cattle Diseases, Heilongjiang Province, Daqing, 163319 China

**Keywords:** *Pasteurella multocida*, OMPH, Ghost, Lytic gene E, Phage PhiX174 RF1, *Escherichia coli*

## Abstract

*Pasteurella multocida* is a pathogen that can infect humans and animals. A ghost is an empty bacterial body devoid of cytoplasm and nucleic acids that can be efficiently presented by antigen-presenting cells. To study a novel ghost vector vaccine with cross-immune protection, we used bacteriophage PhiX174 RF1 and *Pasteurella multocida* standard strain CVCC393 as templates to amplify the split genes E and OmpH to construct a bidirectional expression vector E’-OmpH-pET28a-ci857-E. This is proposed to prepare a ghost *Escherichia coli* (engineered bacteria) capable of attaching and producing *Pasteurella multocida* OmpH on the inner membrane of Escherichia coli (BL21). The aim is to assess the antibody levels and the effectiveness of immune protection by conducting a mouse immunoprotective test. The bidirectional expression vector E’-OmpH-pET28a-ci857-E was successfully constructed. After induction by IPTG, identification by SDS-PAGE, western blot, ghost culture and transmission electron microscope detection, it was proven that the *Escherichia coli* ghost anchored to *Pasteurella multocida* OmpH was successfully prepared. The immunoprotective test in mice showed that the antibody levels of *Pasteurella multocida* inactivated vaccine, OmpH, ghost (aluminum glue adjuvant) and ghost (Freund’s adjuvant) on day 9 after immunization were significantly different from those of the PBS control group (*P* < 0.01). The immune protection rates were 100%, 80%, 75%, and 65%, respectively, and the PBS negative control was 0%, which proved that they all had specific immune protection effects. Therefore, this study lays the foundation for the further study of ghosts as carriers of novel vaccine-presenting proteins.

## Introduction

Pasteurellosis is a highly contagious and potentially fatal infectious disease in cattle caused by *Pasteurella multocida (P. multocida)*. It is characterized by sudden onset, high fever, and widespread infection, resulting in significant damage. Commonly referred to as bovine hemorrhagic sepsis, this disease poses a significant threat to the global cattle industry. *Pasteurella multocida* is a ubiquitous Gram-negative bacterium affecting many host species worldwide. Although the clinical manifestations vary, pasteurellosis mainly causes pneumonia and haemorrhagic septicemia [1]. Humans suffer from pasteurellosis [2], predominantly caused by infection from animal scratches and bites, especially in the elderly and children. Outbreaks of this disease lead to economic losses in meat and dairy-related industries and pose safety risks to human health. Vaccination for HS is the only feasible way to prevent the disease [3, 4]. So far, various modern vaccines, including recombinant vaccines, have been developed as veterinary vaccine candidates for the prevention of HS [5]. However, due to the low cross-immunity protection, various factors lead to the destruction of the conformation of bacterial antigenic determinants, significantly affecting vaccines’ immune effect. Therefore, it is imperative to seek broad-spectrum protection HS vaccines with durable immunity [6].

The ghost is the lytic protein expressed by the phage lytic gene E in Gram-negative bacteria, which causes pores of 40–200 nm in the inner and outer membranes of the bacteria, predisposing the bacteria contents to overflow due to the action of osmotic pressure [7]. Because of their excellent immune effect and low cost, Bacterial Ghost can be used as a delivery vehicle for protein subunit vaccines [8–12]. Depending on where the protein is expressed, ghost protein expression can be achieved in the following ways: (1) The target protein is expressed on the outer membrane by its fusion expression with the membrane protein of the host bacteria [13–15]. Jechlinger W et al. successfully used *Escherichia coli (E. coli)* OmpA and HBV core antigen gene fusion to prepare a protein subtype unit vaccine ghosts [16]. (2) The target protein is expressed on the intracellular membrane by fusing it with the membrane anchor sequence. There have been successful cases showing that the ghost produced by the fusion expression of the anchor gene and the target protein can induce an immune response [17]. (3) The target protein is expressed in the periplasmic space by fusion expression of the target protein and the periplasmic space protein [18]. Many cloud-like self-assembled superstructures are formed in the periplasm [19, 20]. When bacteria are lysed, their inner and outer membranes are fused, the periplasmic space is closed, and the expressed proteins remain in the periplasmic cavity without overflowing with the lysis of bacteria.

*Pasteurella multocida* outer membrane protein H (OmpH) is considered an immunodominant porin, and the OmpH protein of each serotype has high homology. The OmpH protein can induce high levels of protective antibodies, which are potential targets for vaccine candidates [21, 22]. The study of Lu and others has shown that purified OmpH can induce a 100% protection rate comparable to that of whole bacteria [23]. The OmpH protein and the whole cell can generate high levels of antibodies, with the OmpH protein having high homology with the P2 porin of *Haemophilus influenzae*. The study by Vasfr and others has proven that OmpH has a high degree of homology in *Pasteurella multocida* and found that its polyclonal antibody can react with the microporin of *Haemophilus paraavium* [24]. Passive immunization of mice with a monoclonal antibody against this protein can also prevent infection in mice. In addition, Sezer Okay et al. demonstrated the immunogenicity of a protein vaccine created using the outer membrane protein OmpH in mice [25].

Therefore, this study intends to prepare *Escherichia coli* ghosts anchored to *Pasteurella multocida* OmpH, and applied to the immunoprophylaxis of bovine colibacillosis and pasteurellosis. The ghost platform of bidirectional expression vector system was initially established and prepared to provide technical support for the development of novel efficient dairy cow vaccines and other *Escherichia coli* ghost vector delivery systems.

## Materials and methods

### Strains and reagents

The standard strain CVCC393 of *Pasteurella multocida* was purchased from the China Culture Storage Center. *Escherichia coli* DH5α, *E. coli* host strain BL21 (DE3), host strain Rossta (DE3), and prokaryotic expression vector pET28A were all preserved in the laboratory at the Heilongjiang Provincial Engineering Research Center for Prevention and Control of Cattle Diseases. pBV220 (containing λpL/pR promoter and repressor protein cI857) was a gift from Dalian Sanyi [26]. PhiX174 RF1 and pMD18-T were purchased from Dalian TAKALA Biological Company. Five to eight weeks-old healthy SPF clean-grade Kunming female mice were purchased from Changchun Experimental Animal Center, Jilin. IPTG was purchased from Beijing Kangwei Century Biotechnology Co., Ltd. Restriction endonucleases and T4 DNA Ligase were purchased from the United States Fermentas (MBI) company. BHI was purchased from Becton Dichinson (BD). Gel Extraction Kitd2500-01, gel recovery kit and Plasmid Mini Kitd6943-01 were purchased from OMEGA Co., Ltd. Goat anti-mouse IgG-HRP and DAB substrate chromogenic solution were purchased from Beijing Zhongshan Golden Bridge Bioengineering Co., Ltd. IgG production was measured using commercial ELISA kits (USCN Life Science, Wuhan, China). SPF mice were purchased from Beijing Weitong Lihua Laboratory Animal Technology Co., Ltd (Beijing, China) (6–8 weeks old, 18–22 g). All mice were maintained in an IVC stand-alone ventilated feeding system and handled strictly with the guidelines and protocols approved for these experiments by the Management Committee of the Experimental Animal Center of Heilongjiang Bayi Agricultural University.

### Construction of E-PMD18-T vector and identification of positive clones

Using Oligo 6.0 and primer 5.0 software, primers were designed according to the coding sequence of phage PhiX174 cleavage gene E (GenBank: NC-001422) registered on GenBank. The restriction enzyme site *EcoRI* was introduced at the 5’ end of the upstream primer, and the *Sal I* restriction enzyme site was introduced at the 5’ end of the downstream primer. The Shanghai Biotechnology Synthesis Department synthesized the primers. E Forward primer (F1): 5’-CGCGAATTCATGGTACGCTGGACTTTGTG-3’. The underlined sequence is the restriction enzyme cleavage site EcoRI cleavage site. E Reverse primer (R1): 5’-ACGCGTCGACTCACTCCTTCCGCACGTAAT-3’. The underlined sequence is the restriction enzyme cleavage site Sal I cleavage site. The reaction conditions were as follows: pre-denaturation at 95 °C for 5 min, denaturation at 94 °C for 50 s, annealing at 61 °C for 30 s, extension at 72 °C for 30 s, and final extension at 72 °C for 10 min. OMEGA company’s gel extraction kit (D2500-01) was then used to recover the target fragment E. The gene E was linked to pMD18-T and transformed into *E. coli* DH5α competent cells. Sequencing analysis was carried out after restriction enzyme digestion and PCR identification.

### Construction and expression of prokaryotic expression vector pbv220-E

The correctly sequenced E-pMD18-T and pBV220 plasmids were double digested with restriction enzymes *EcoRI* and *Sal I* and recovered by gel. This was followed by ligation using the T4 ligase of MBI and then transformed into *E. coli* DH5α competent cells and the recombinant vector pBV220-E were identified using the above method. The positive recombinant plasmid was named pBV-E.

The pBV220-E/DH5α strain was properly identified and induced for expression in a medium containing 100 ug/ml ampicillin. Before and after induction, a small portion of the bacterial solution was taken and diluted with sterile PBS to achieve the same multiplicity. Subsequently, the diluted solution was spread on LB solid medium and incubated at 28 °C for 48 h. The number of viable bacteria (CFU/mL) and the lysis rate were determined. The lysis rate was calculated using the formula: Lysis rate = (1-CFU after induction/CFU before induction) × 100% [27]. Next, the culture was centrifugated at 3500 r/min at room temperature for 15 min. The supernatant was discarded, and the resulting precipitate was sent to the Electron Microscopy Group at the Harbin Veterinary Research Institute for observation using a transmission electron microscope.

### Construction of recombinant plasmid E’-OmpH-pET28a

The first step involved the preparation of genomic DNA from *Pasteurella multocida*. Subsequently, two pairs of overlapping complementary primers were designed based on the constructed recombinant plasmids pBV-E and Omph (GenBank: HM582886.1). A *BamH I* restriction site was introduced into the 5’ end of the upstream primer F1 (underlined), and the 3’ end of the downstream primer K2 introduced an *XhoI* restriction enzyme site (underlined). Recombinant prokaryotic expression plasmid pBV-E was used as a template, with primers F1 and F2 amplifying the anchor gene E. Furthermore, the *Pasteurella multocida* genome was used as a template, with primers K1 and K2 to amplify the OmpH gene, and then primers F1 and K2 were used to amplify E’- OmpH fusion gene. The nucleotide sequences of the primers are as follows: E’: Forward primer F1: 5’-CGCGGATCCATGGTACGCTGGACTTTGTG-3’. Reverse primer F2: 5’-CCGCCACCGCTGCCACCCCCGCCGACGCTCGACGCCATTAATAAT-3’. OmpH: Forward primer k1: 5’-TGGCAGCGGTGGCGGGGGATCAGCAGTCGCAGCAGTAGCAGC-3’. Reverse primer k2: 5’-CCGCTCGAGTTAAACGACATTGCCTTTTGTTGTT-3’.

### Amplification of E’ and OmpH genes

To amplify the E’ gene, pBV-E was utilized as a template, and primers F1 and F2 were employed. On the other hand, to amplify the OmpH gene, the genome of Pasteurella multocida CVCC393 was used as a template, along with primers K1 and K2. Conditions for E’ reaction include pre-denaturation at 95 °C for 5 min, denaturation at 95 °C for 30 s, annealing at 62 °C for 30 s, extension at 72 °C for 30 s, a total of 30 cycles, and a final extension at 72 °C for 10 min. Conditions for Omph reaction include pre-denaturation at 95 °C for 5 min; 30 cycles of denaturation at 95 °C for 30 s, annealing at 58 °C for 30 s, and extension at 72 °C for 90 s; final extension at 72 °C for 10 min. The amplified products were analyzed by electrophoresis in 1% agarose gel.

### Construction of E’-OmpH fusion gene expression vector

The PCR products of the two genes above were recovered and purified. The recovered products of genes E’ and OmpH were then employed as templates, while F2 and K1 primers were used to perform overlap PCR to splice and amplify the E’-OmpH gene fragment. The expected size of the resulting fragment is 1149 bp. The PCR reaction conditions were as follows: pre-denaturation at 95 °C for 5 min, denaturation at 95 °C for 30 s, annealing at 58 °C for 30 s, extension at 72 °C for 1 min for 30 s, a total of 30 cycles, and a final extension at 72 °C for 10 min. The amplified products were analyzed by electrophoresis in 1% agarose gel.

### Construction and expression of bidirectional expression vector E’-OmpH-pET28a-ci857-E

According to the published pBv220 and E gene sequences, primers were designed using Primer 5.0 and Oligo 6.0 software. A SgrAI restriction endonuclease site (underlined part) was introduced at the 5’ end of the upstream primer. In comparison, *SphI* restriction was introduced at the 5’ end of the downstream primer endonuclease site [26, 28]. Shanghai Sangon Bioengineering Technology Service Co., Ltd, synthesized primers. The nucleotide sequences of the primers are as follows: ci857A Forward primer: 5’-TACATACrCCGGyGTCAGCCAAACGTCTCTTC-3’ (SgrAI). ci857B Reverse primer: 5’-ACATGCATGCGTTTGTAGAAACGCAAAAAGG-3’ (SphI). The size of the PCR-amplified nucleic acid fragment was 1888 bp.

### Amplification of ci857-E gene in lysis system

The ci857-E-T1T2 gene was amplified with pBv220-E as a template and ci857A and ci857B as primers. The PCR reaction system was as follows: pre-denaturation at 95 °C for 5 min, denaturation at 95 °C for 30 s, annealing at 58 °C for 30 s, extension at 72 °C for 1 min, a total of 30 cycles, and a final extension at 72 °C for 10 min. PCR amplification products (5 µL) were used for electrophoresis analysis in 1% agarose gel.

### Construction and identification of bidirectional expression vector

The PCR product of the cleavage system ci857-E gene was recovered and purified. The recovered target fragment ci857-E and the recombinant vector E’-OmpH-pET28a were digested with double enzymes *SgrAI* and *SphI*, and the target fragment and vector were recovered, respectively. Ligation was done overnight at 22 °C using T4 DNA Ligase. The ligated products were transformed into *E. coli* BL21 (DE3) and *E. coli* rossta (DE3) competent cells, screened for kanamycin resistance. Positive cloned plasmids were extracted and identified by PCR and double digestion was performed with *SgrAI* and *SphI*. The recombinant clones identified as positive were sent to Shanghai Sangon Biotechnology Co., Ltd. for sequence determination, and the recombinant plasmid with correct sequencing was named E’-OmpH-pET28a-ci857-E. The protein of E’-OmpH was purified by nickel column affinity chromatography kit and analyzed by western blot.

### Drawing of cleavage curve of the bidirectional expression vector and transmission electron microscopy of ghosts

IPTG induced E’-OmpH-pET28a-ci857-E/BL21 at 28 ℃ for 5 h, 37 ℃ for 5 and 12 h, then the OD 600 value was detected, and the lysis curve was drawn. The induced E’-OmpH-pET28a-ci857-E/BL21 was sent to the electron microscopy group of Harbin Veterinary Research Institute for TEM section images.

### Experimental animal grouping and immunization procedure

Mice were randomly divided into 6 groups, with 20 mice in each group: PBS control, OmpH protein, *Escherichia coli* ghost (aluminum glue), *Pasteurella multocida* inactivated vaccine, *Escherichia coli* ghost (Freund’s adjuvant) and minimum lethal dose test groups, respectively. The immunization doses of each group are shown in Table 1. ① OmpH protein group dialysis bag was concentrated to 200 ug/100 µL, a required concentration for immunization, mixed with Freund’s adjuvant in equal volume, and emulsified to prepare oil emulsion subunit vaccine. Freund’s complete adjuvant was used for the first immunization, and Freund’s adjuvant was used for the second immunization. The incomplete adjuvant was 100 µL per mouse per immunization. ② *Escherichia coli* ghost group (Freund’s adjuvant), 200 µg/100 µL per mouse was mixed with Freund’s adjuvant in an equal volume, emulsified to prepare oil emulsion subunit vaccine, and Freund’s complete adjuvant was used for the first immunization. Freund’s incomplete adjuvant was used for secondary immunization. Each mouse was inoculated with 100 µg dry weight of the *Escherichia coli* ghost group (Freund’s adjuvant). ③ *Escherichia coli* ghost group (aluminum glue group) was mixed according to the ratio of ghost and aluminum glue in the proportion of 1:3, and each mouse was inoculated with the *Escherichia coli* ghost group with a dry weight of 100 ug. ④ PBS control group was injected with 100 µL of PBS.

Before immunization, tail-cut blood was collected, and the serum was separated and used as a negative control serum. An intraperitoneal injection followed the first intraperitoneal immunization (on day 0) for the second immunization (on day 3), and blood was collected from the tail vein of mice one week later. Two weeks later (17 days), the mice were injected intraperitoneally three times for immunity, and blood was collected from the tail vein of the mice 9 days after the three times of immunization, while the serum was separated for antibody detection. Euthanasia was carried out by pumping 100% CO_2_ into a sealed chamber at 50% VDR/min, per approved IACUC protoncol. That exposure times of at least 5 min in carbon dioxide were required to ensure irreversible euthanasia of mice [29–31].

### Detection of serum IgG levels by ELISA

First, we optimized the working concentrations of antigen, serum, and secondary antibodies. Nickel column purified E’-OmpH was concentrated at 200 µg/100 µL. The antigen was diluted with carbonate buffer to a concentration of 20 µg/100 µL; it was coated overnight at 4 °C and incubated with serum and secondary antibody the next day. The four dilution concentrations of serum are 1:200, 1:400, 1:800, and 1:1600. The dilutions of enzyme-labeled secondary antibodies were 1:3000, 1:6000, 1:12000, and 1:14000, respectively. After the TMB color was developed, it was terminated with concentrated sulfuric acid, determined at an OD value of 450, and the results were analyzed. At the same time, blank, positive and negative wells were set as controls. The indirect ELISA method detected the IgG level in mouse serum according to the manufacturer’s instructions.

### Challenge protection test

First, the minimum lethal dose was determined, and the mice were randomly divided into 7 groups, with 6 mice in each group. The first group was intraperitoneally injected with 1 mL of *Pasteurella multocida* bacteria solution with a concentration of 1 × 10^10^ CFU/mL; the second group was injected with 0.80 mL; the third group with 0.60 mL, the fourth group with 0.40 mL, and the fifth group with 0.20 mL. The sixth group was injected with 0.10 mL, and the seventh group with PBS as a control. Mice death was observed and recorded for 7 consecutive days.

The challenge dose for the mice in each group was determined based on the minimum lethal dose (MLD) of Pasteurella multocida. Two weeks after the three immunizations, 10 mice in each group were intraperitoneally injected with the challenge dose set at 1 MLD. The death of the mice was observed daily within one week after the challenge. The mice that died from the challenge were taken for autopsy, their lesions were observed, and their organs (liver, spleen and lymph nodes) were taken under sterile conditions for bacterial isolation and identification.

## Results

### Successful preparation of ***Escherichia coli*** ghosts

**Fig. 1:**
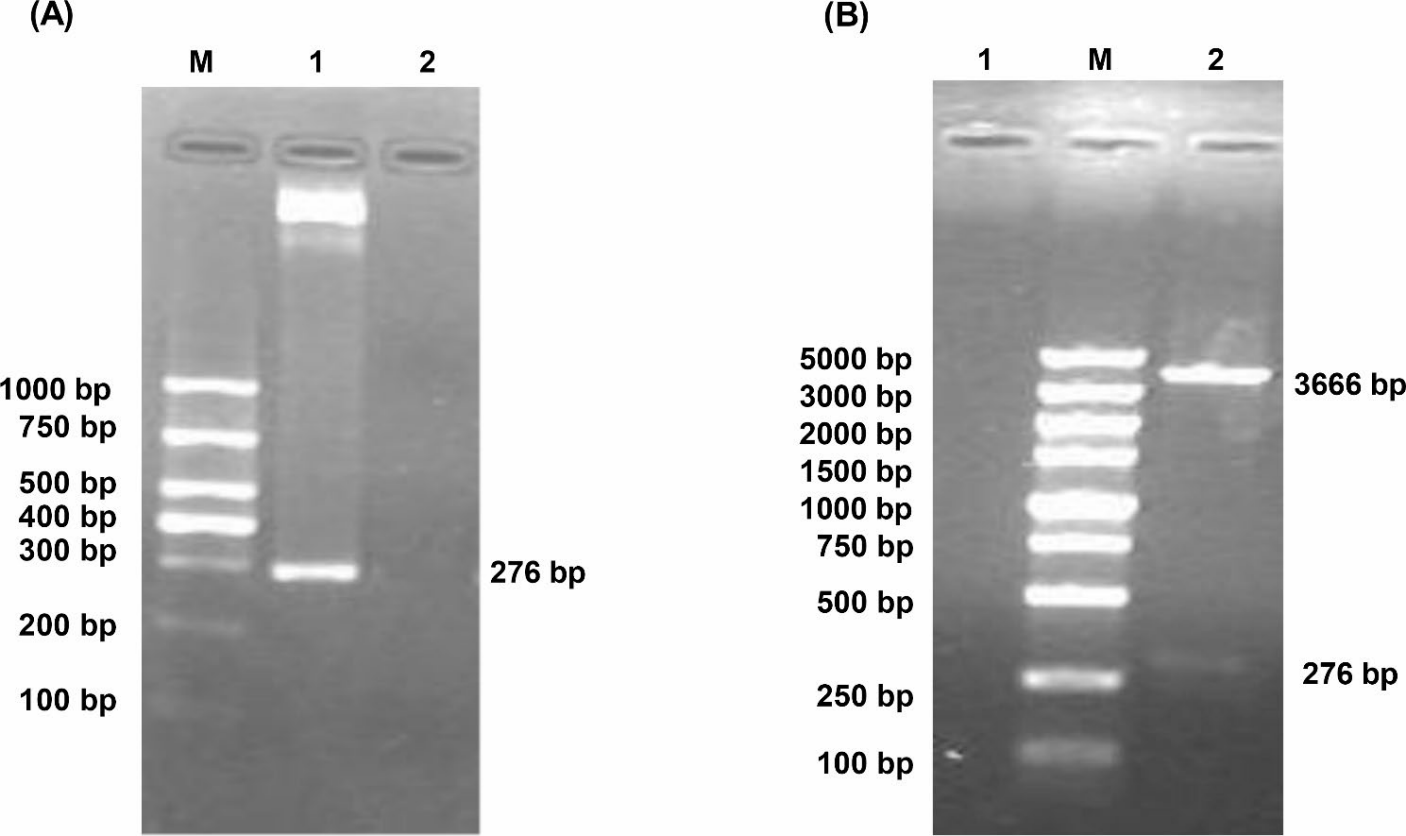
Identification results of gene E and recombinant vector pBV-E. **A**: PCR amplification results of gene E. M: DL1000 DNA Marker; 1: The Amplification of gene E; 2: Negative Control. **B**: PBV-E digestion results. 1: Negative Control; M: DL5000 DNA Marker ; 2: double digested product of recombinant plasmid pBV-E.

PCR amplified the E gene of phage PhiX174 RF1 with the designed primers. The PCR products were electrophoresed on a 1.5% agarose gel, and specific DNA bands were seen, consistent with the expected size (E: 276 bp) (Fig. 1(A)). The recombinant plasmid pBV-E was double digested with the endonucleases *EcoRI* and *Sal I* at both ends of the E gene to obtain two fragments, the first fragment was about 3666 bp, and the second fragment was approximately 276 bp, which are respectively the same as the vector pBV and the insert fragment gene E size (Fig. 1(B)). Alignment of the recombinant bacteria containing the pBV220-E plasmid after sequencing was done using NCBI BLAST. The full-length gene sequence measured was 276 bp, compared with the phage PhiX174 gene E sequence published on GenBank; the homology was 100% (276/276). The nucleotide sequence of the gene E obtained by sequencing was translated into an amino acid sequence, which was compared with the amino acid sequence of the protein E on GenBank, and the homology was 100% (91/91).

**Fig. 2:**
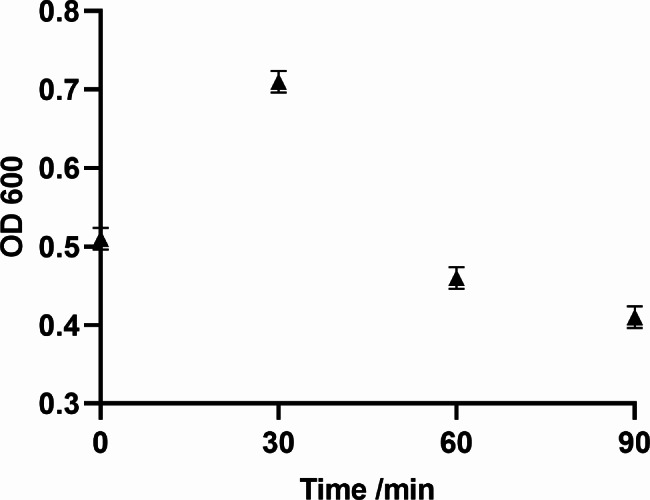
The results of pBV-E *Escherichia coli* ghost lysis

**Fig. 3:**
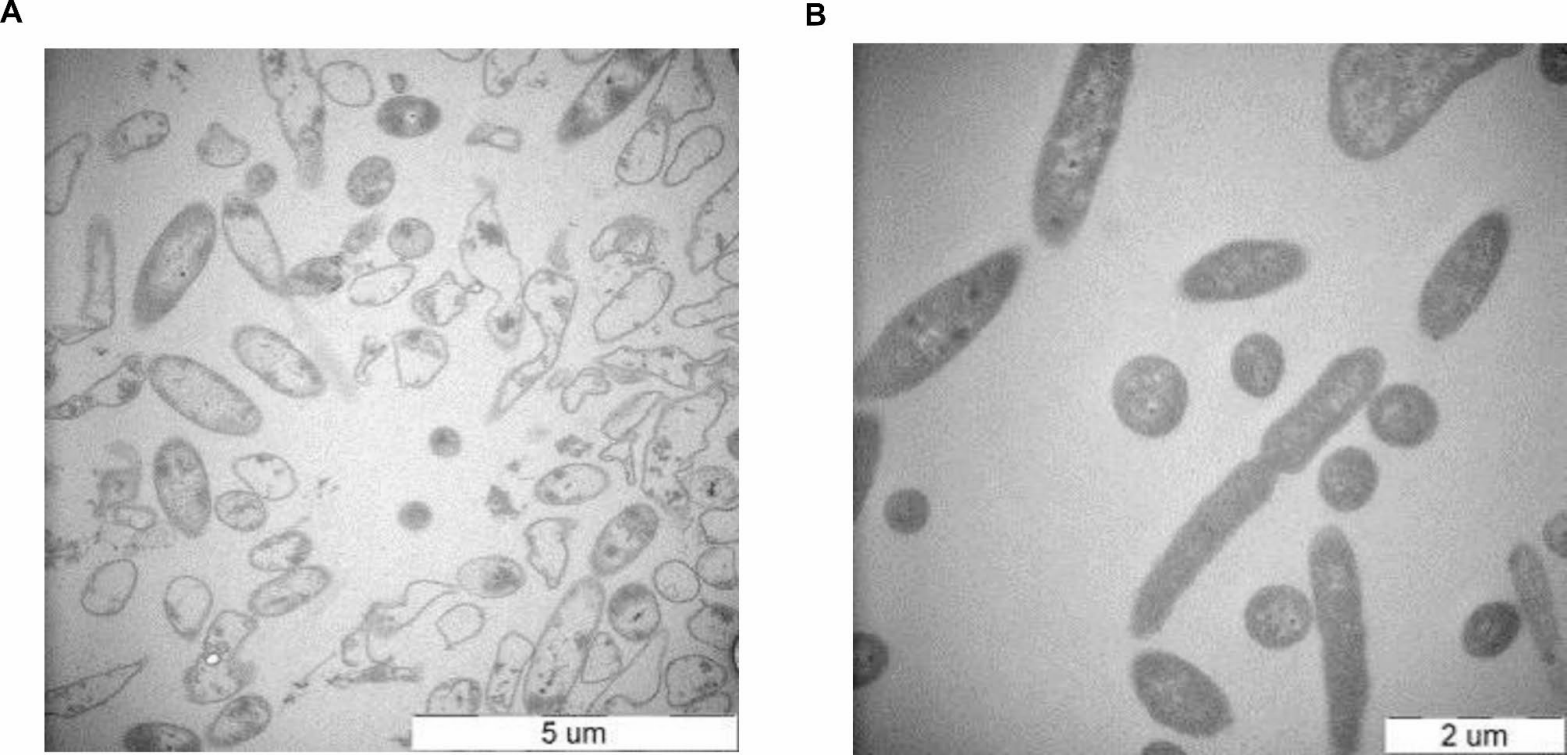
Transmission electron microscope results of *Escherichia coli* ghost pBV-E/DH5α. A: pBV-E/DH5α ghost; B: DH5α did not lyse bacteria

We found that the OD 600 of pBV-E/DH5α slightly increased upon heat induction for about half an hour; it decreased and then rose again after 2 h, and the lower the bacterial concentration during induction, the better the lysis effect (Fig. 2). The bacterial solution before and after induction was diluted to 10^− 6^ and 10^− 2^ times with LB liquid medium, respectively, and plated. Each sample was repeated three times and incubated at 28 ℃ for 48 h, and the calculated lysis rate was 99.72%. In addition, through the transmission electron microscope image of *Escherichia coli* ghost pBV-E/DH5α, it was found that the bacterial ghost was an empty bacterial shell without content under the electron microscope (Fig. 3).

### The recombinant plasmid E’-OmpH-pET28a was constructed

**Fig. 4:**
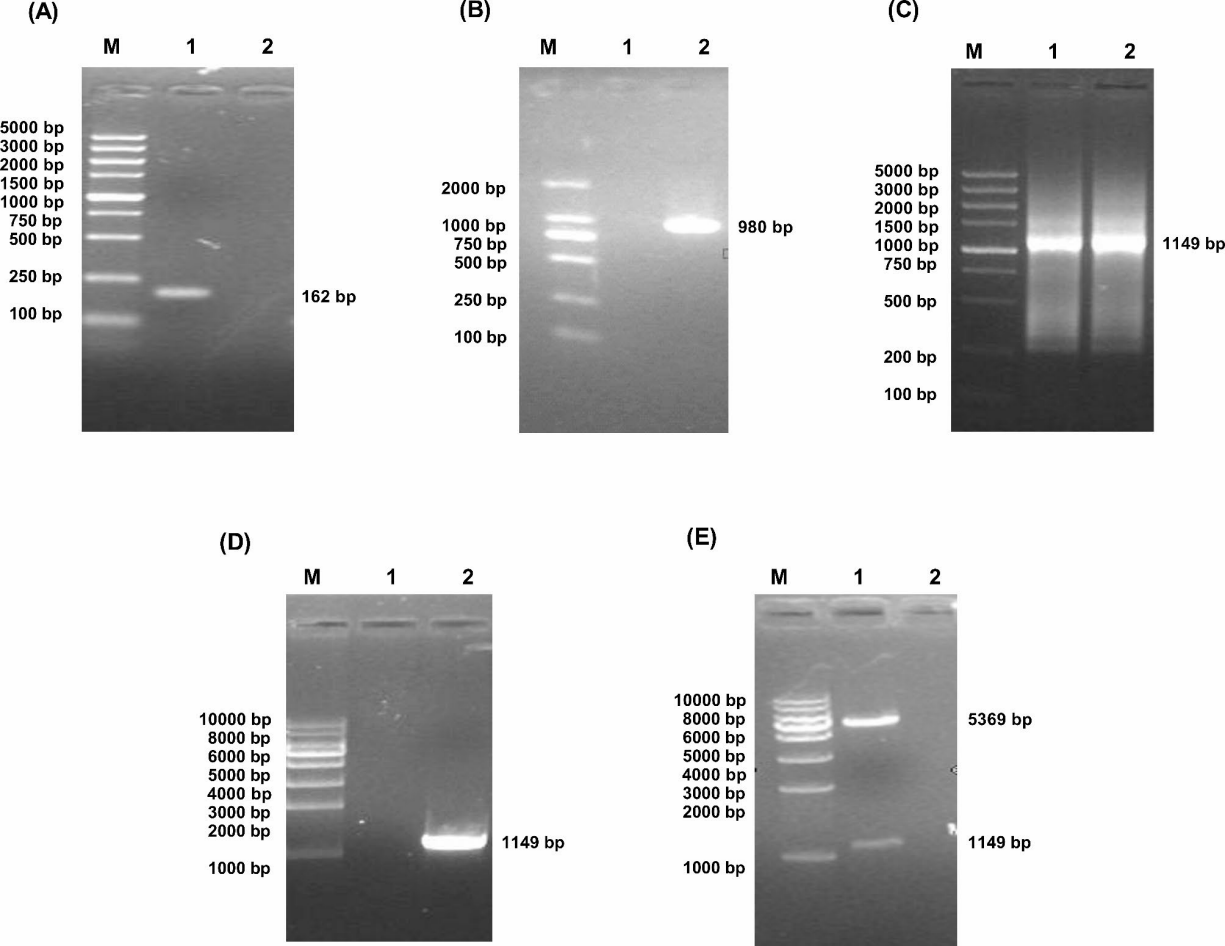
**E’-Omph fusion expression identification results.** A: PCR amplification results of E’ gene. M: DL5000 DNA marker; 1: Gene E’; 2: Negative Control. B: PCR amplification results of gene OmpH. M: DL2000 DNA Marker; 1: Negative Control; 2: OmpH gene. C: PCR amplification results of gene E’-Omph. M: DL5000 DNA Marker; 1–2: PCR Amplification of E’-Omph combined gene. D: PCR Identification Results of Recombinant Plasmid E’-Omph-pET28a. M: DL10,000 DNA Marker; 1: Negative Control; 2: PCR product of E’-OmpH-pET28a. E: Identification results of recombinant plasmid E’-Omph-pET28a. M: DL10,000 DNA Marker; 1: E’-Omph-pET28a digested by *BamH I* and *XhoI*; 2: Negative Control

The E’ gene of pBV-E and the OmpH gene of the *Pasteurella multocida* genome were amplified by PCR with the designed primers. The PCR products were subjected to 1% agarose gel electrophoresis,. Specific DNA bands were seen, consistent with the expected fragment size (E ‘: 162 bp, OmpH: 980 bp) (Fig. 4A-B). The E’-Omph gene was amplified by the overlap PCR method using overlapping primers. The PCR product was electrophoresed on a 1% agarose gel, and a specific DNA band was seen, consistent with the expected size (1149 bp) (Fig. 4C). After the above PCR product was recovered and purified, it was connected to the pET28a expression vector and transformed into BL21 (DE3) competent cells. PCR and double digestion identified the recombinant E’-OmpH-pET28a plasmid. After *BamHI* and *XhoI*, a 5369 bp vector fragment and an 1149 bp destination fragments are visible (Fig. 4E). The 1149 bp target fragment was also seen in PCR amplification (Fig. 4D).

NCBI BLAST was used for alignment after sequencing the recombinant bacteria containing the E’-Omph-pET-Ci857-E plasmid. The full-length gene sequence measured was 1149 bp, consistent with the E’ and OmpH gene sequences published on GenBank. The alignment was performed, the homology was 99.9% (1148/1149), and 1 gene difference existed in the OmpH gene. The nucleotide sequence of the E’-Omph gene obtained by sequencing was translated into an amino acid sequence, which was compared with the amino acid sequences of E’ and OmpH proteins in the GenBank, and the homology was 100% (383/383).

### The bidirectional expression vector E’-OmpH-pET28a-Ci857-E was constructed

**Fig. 5:**
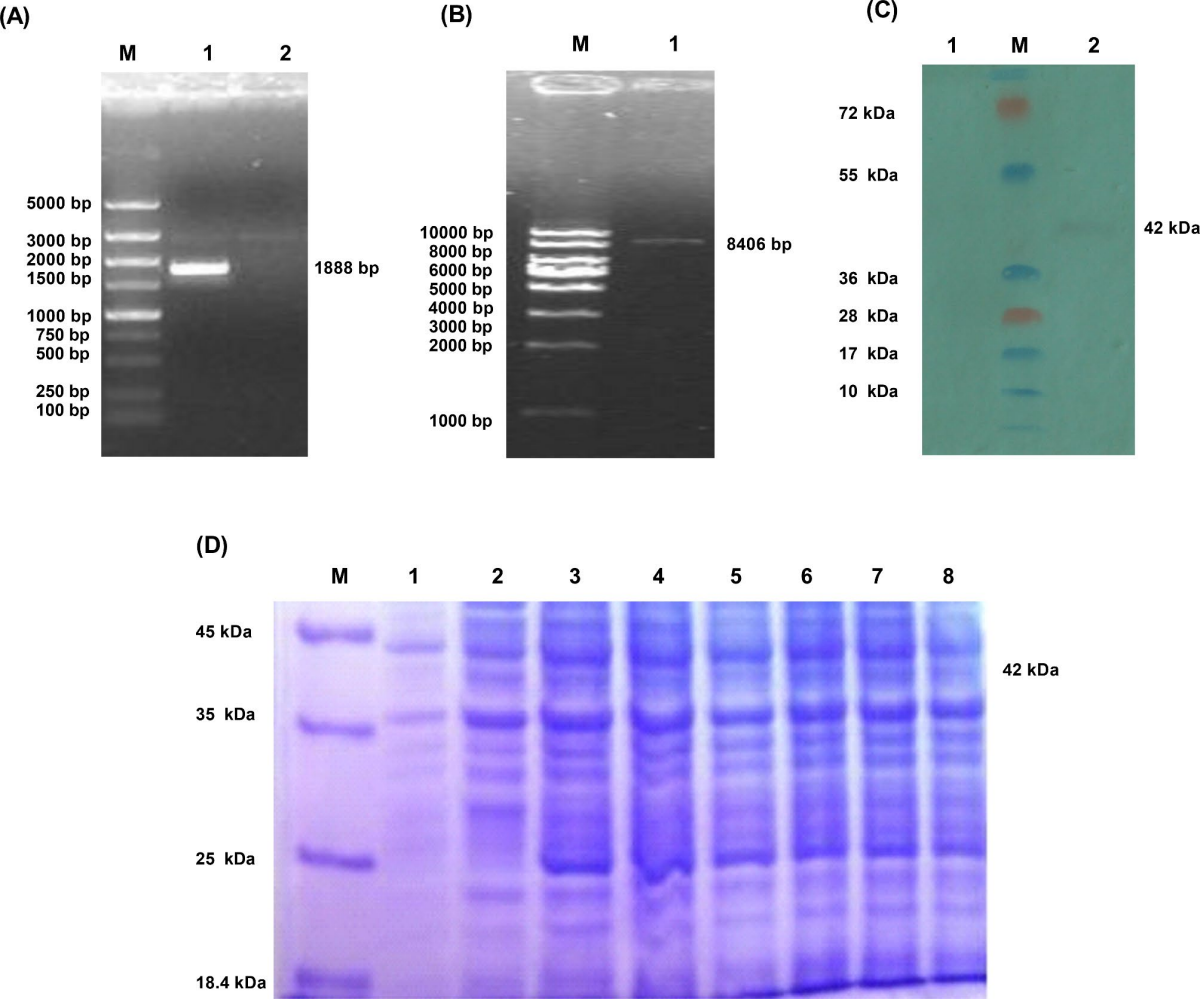
Results of ci857-E gene and recombinant protein. **A**: PCR amplification results of ci857-E gene. M: DL5000 DNA Marker; 1: ci857-E gene; 2: Negative control. **B**: Identification results of recombinant plasmid E’-OmpH-pET28a-ci857-E. M: DL10,000 DNA Marker; 1: pET28a-Ci857-E digested by *BamHI*. **C**: Western blot analysis of recombinant protein E’-OmpH. M: Prestained protein marker; 1: Negative Control; 2: Anti-His antibody as the first antibody. **D**: SDS-PAGE results of recombinant fusion protein expression. M: Marker; 1 is the *E. Coli* BL21 (E’-Omph -pET28a-ci857-E) before induce; 2 is first induced *E. Coli* BL21(E’-Omph -pET28a-ci857-E) by IPTG for 12 h in 28 ℃ then induced by shifting temperature at 42 ℃; 3, 4, 5 is the induced *E. Coli* BL-21 (E’-Omph -pET28a-ci857-E) by IPTG for 5 h in 37 ℃, 12 h in 28 ℃, 5 h in 28 ℃; 6, 7, 8 is the induced *E. Coli* Rossta (E’-Omph -pET28a-ci857-E) by IPTG for 5 h in 37 ℃, 12 h in 28 ℃, 5 h in 28 ℃

The ci857-E gene of the designed primer pBV-E was used for PCR amplification. The PCR products were electrophoresed on 1% agarose gel. Specific DNA bands were seen, consistent with the expected size (1888 bp) (Fig. 5A). After recovery and purification of the above PCR product, it was connected to the pET28a expression vector and transformed into *E. coli* BL21 (DE3) competent cells. The recombinant E’-OmpH-pET28a-ci857-E plasmid was identified by *BamHI* digestion, and the size was around 8406 bp (Fig. 5B).

**Fig. 6:**
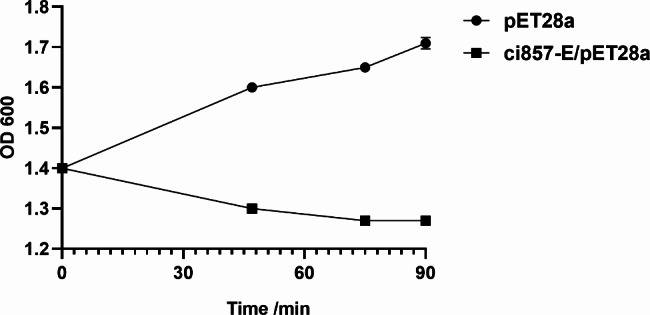
E’-OmpH-PET28a-ci857-E/BL21 cleavage results

**Fig. 7:**
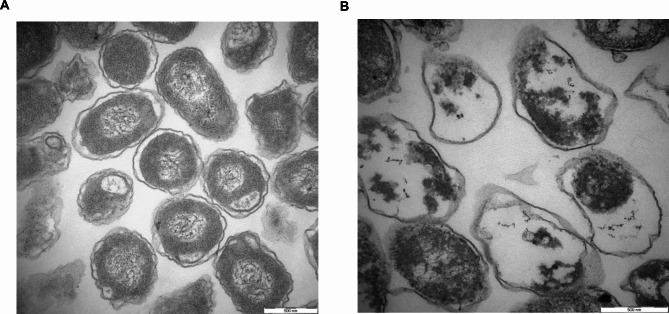
Results of *Escherichia coli* Ghost of anchoring bovine *Pasteurella multocida* OmpH.

The recombinant expression vectors E’-Omph-pET28a-ci857-E/Bl21(DE3) and E’-Omph-pET28a-ci857-E/Rossta (DE3) were expression-induced, identified by SDS-PAGE. The result showed that the vector E’-Omph-pET28a-ci857-E was induced with a specific band at 42 kDa, consistent with the expected protein size (Fig. 5C). The purified recombinant protein E’-Omph-pET28a was transfected into a membrane, the mouse anti-his-tag protein monoclonal antibody was used as the primary antibody, and the goat anti-mouse IgG antibody was used as the secondary antibody (Fig. 5D). When IPTG was induced at 28 °C for 5 h, the cleavage efficiency remained when the OD 600 reached 1.3. The OD 600 of pBV-E/DH5α increased slightly after induction for about half an hour, then decreased for 2 h and then rose again, while E’-OmpH- OD value of PET28a-ci857-E/BL21 did not increase when the temperature was induced for about half an hour. In contrast, pET28a/BL21 maintained an increasing OD 600 value throughout the process (Fig. 6). *E. coli* ghost anchored to *Pasteurella multocida* OmpH (Fig. 7).

### Immune effect evaluation

**Fig. 8:**
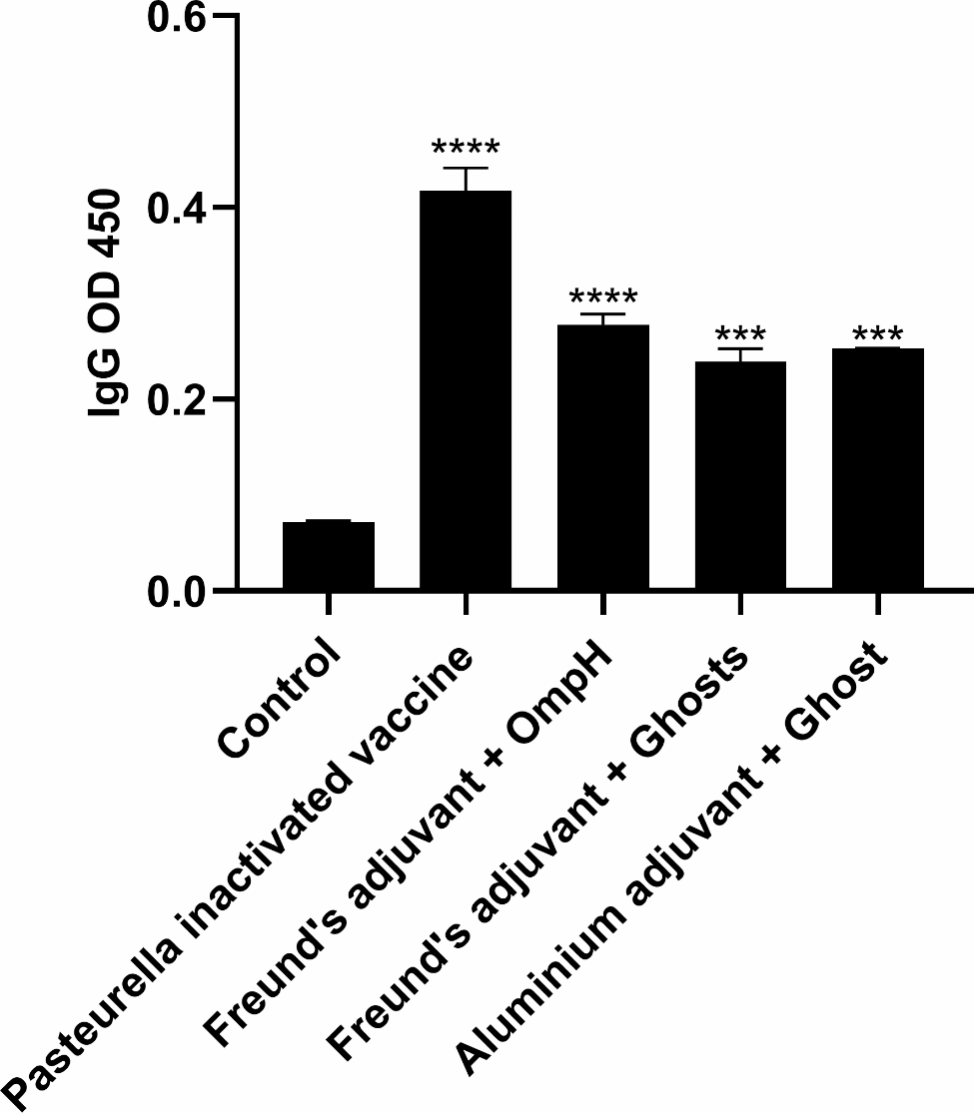
The ebb and flow level of serum IgG antibodies in mice

The average OD 450 of positive and negative serum were 0.434 and 0.075, respectively, with these average values being the largest at this time (Table 1). We detected the immune serum according to the optimized amount of primary and secondary antibodies. Each serum was diluted at 1:800 and the enzyme-labeled secondary antibody at 1:3000 for the indirect ELISA test. IgG antibody levels were detected 9 days after three vaccinations. The serum-specific IgG levels of *Pasteurella multocida* inactivated vaccine, OmpH immunization group, ghost group containing Freund’s adjuvant, and ghost group containing aluminum glue adjuvant were significantly higher than those of the PBS group (*P* < 0.01). The antibody level of the *Pasteurella multocida* inactivated vaccine was significantly higher than other immunization groups. The P/N value of each immunization group was > 2.1, showing immune protection (Fig. 8).

**Table 1:** Detection of serum IgG levels in mice by ELISA. EP: Enzyme primary antibody. ES: Enzyme-labeled secondary antibody. +: Positive serum

EP	ES1/3000			ES1/6000			ES1/12,000			ES1/24,000		
200+	1.328	1.431	1.754	0.618	0.566	0.632	0.124	0.119	0.09	0.059	0.063	0.07
200-	0.915	1.034	0.937	0.215	0.215	0.222	0.068	0.064	0.093	0.052	0.06	0.061
400+	1.527	1.22	1.153	0.221	0.214	0.214	0.076	0.064	0.059	0.059	0.073	0.058
400-	0.453	0.42	0.358	0.121	0.128	0.124	0.069	0.066	0.062	0.069	0.073	0.061
800+	0.411	0.42	0.473	0.057	0.040	0.064	0.052	0.049	0.048	0.064	0.052	0.055
800-	0.079	0.073	0.073	0.042	0.052	0.048	0.084	0.05	0.056	0.061	0.059	0.054
1600+	0.199	0.217	0.245	0.099	0.091	0.141	0.052	0.048	0.052	0.058	0.048	0.056
1600-	0.052	0.054	0.068	0.049	0.052	0.060	0.056	0.049	0.053	0.057	0.072	0.189

The death of the animals was observed for one week after the three-immunization challenge, and the internal organs of the dead mice were collected to isolate pathogenic bacteria. The results showed that *Pasteurella multocida* inactivated vaccine, ghost (aluminum glue adjuvant), OmpH (Freund’s adjuvant), ghost (Freund’s adjuvant) all had specific immune protection effects, and the protection rates were 100%, 80%, 75%, 65%, and the negative PBS control group was 0%. Isolated pathogenic bacteria were aseptically collected from the liver and spleen of the dead mice and identified as Gram-negative *Brevibacterium*, which appeared singly or in pairs and proved to be a virulent strain of *Pasteurella multocida* by biochemical identification.

## Discussion

*Pasteurella multocida* has brought great harm to the aquaculture industry. Still, the pasteurized whole-bacteria inactivated vaccine is serotype-specific because of its numerous serotypes. It cannot play a cross-immune protective response. Studies have shown that OmpH of *Pasteurella multocida* has a cross-immune protective effect, and a good immune protection rate can be obtained by immunizing the animals with OmpH protein [32]. However, the heavy workload of protein purification and the protein may not be well presented. Amara AA et al. have demonstrated that Escherichia coli BL21 (DE3) pLysS (Promega) can be used as a carrier model for the preparation of a general scheme of bacterial shadow (BGs) [33, 34]. And the BL21 (DE3) cell is the first commercially competent cell with a modified LPS (lipid IVA) that does not trigger the cellular endotoxin response. So, we used ghosts as a carrier delivery system, using the natural antigenic determinants of ghosts to easily present complex antigenic determinants to the immune system and targeted delivery of proteins to the body [35].

During the production process, the ghost vaccine is less affected by physical and chemical factors; instead, it maintains the natural structure of the immunogen and can act as an excellent immunogen to protect animals [9, 28]. Ghost vaccines are about to be adopted in various countries as multiple and multivalent vaccines. However, in China, ghost research, especially as a transport carrier, has just started, and many problems still need to be studied and discussed. As an immunogen, the ghost can directly act on the body, and it can also be used as a carrier to deliver proteins, nucleic acids and other drugs to the body, and certain progress has been achieved. Ghosts are used as carriers to provide proteins, mainly in two ways [34]: (1) The purified protein is directly immersed and loaded into the ghost, and the addition of calcein can fuse the membrane sac with the inner film of the ghost to sew it together; (2) The target protein is immobilized and targeted to the bacterial inner membrane by using the anchor protein. In the existing recombinant ghost vaccines, the membrane anchoring sequences used to transmit exogenous subunit proteins mainly include outer membrane anchoring protein OmpA (expressed outside the cell membrane), pericytoplasmic space anchoring protein SbsA (in the periplasmic expression), endomembrane anchoring proteins E’ and L’ (expressed in the intracellular membrane).

We adopted the fusion expression method of inner membrane-anchored protein E’ and *Pasteurella multocida* OmpH because E’ is the 1-54th amino acid on the phage PhiX174 cleavage protein E. The amino acid sequence is precise, and the cleavage protein’s materials are easy to obtain. PCR amplification in E and membrane anchoring of the E’ sequence does not interfere with the correct folding of the target protein. In order to prevent the cells from being lysed prematurely due to read-through during translation, we adopted two independent expression systems, one is the original expression system of pET28a, and the other is a temperature-controlled lysis expression system with the opposite expression direction of pET28a. First, the exogenous gene expression was induced by IPTG for 3–5 h, followed by the heat-induction of bacterial lysis. In this study, the induction method was to add IPTG to the bidirectional expression vector at 28 ℃ for 5–12 h and then rapidly increase the temperature at 42 ℃ for a further 1–2 h period. At the same time, it was necessary to express Pasteurella’s OmpH protein fully and completely lyse the cells. This requires finding out the optimal induction conditions. Our research proves that the bidirectional expression system has an expression effect when induced alone. The bacteria cells were prone to lysis after inducing OmpH expression, and we found that the bacterial cell concentration OD 600 value was about 1.2. The in vivo immune effect proved that the *Escherichia coli* ghost vaccine anchored to *Pasteurella multocida* OmpH had immune protection. In addition, aluminum gel and Freund’s adjuvant mixed with ghosts were used for emulsification immunization.

In summary, we successfully constructed the E’-OmpH fusion gene and temperature-controlled lysis cassette of pBV-E *Escherichia coli* ghost and ankyrin fused with the *Pasteurella multocida* OmpH gene. In addition, our research recorded a breakthrough in preparing a bidirectional expression vector E’-OmpH-pet28a-ci857-E. This confirms the successful expression of the E’-OmpH fusion protein and cleavage gene and the body’s ability to induce the production of specific antibodies and cellular immune responses. Animal experiments proved that bovine-anchored *Escherichia coli* ghosts could resist *Pasteurella multocida* infection and improve immune protection. Therefore, the *Escherichia coli* ghosts prepared by this method can be applied to the immune prevention of bovine colibacillosis and pasteurellosis. At the same time, other *Escherichia coli* ghosts can be prepared by this method, which can be widely used in the veterinary field.

## Data Availability

The data supporting the findings are included in the manuscript. The datasets analysed during the current study are available in the GenBank repository [GenBank: NC-001422.1; GenBank: HM582886.1; GenBank: WP_083008085.1; GenBank: NP_040709.1]. Additional data and materials are available upon request from the corresponding author.
